# Autophagy in proteostasis and aging in *Caenorhabditis elegans*

**DOI:** 10.1016/j.cstres.2025.100115

**Published:** 2025-09-18

**Authors:** Caitlin M. Lange, Ryo Higuchi-Sanabria, Caroline Kumsta

**Affiliations:** 1 Graduate School of Biomedical Sciences, Sanford Burnham Prebys Medical Discovery Institute, La Jolla, CA 92037; 2 Center for Cardiovascular and Muscular Diseases, Sanford Burnham Prebys Medical Discovery Institute, La Jolla, CA 92037; 3 Leonard Davis School of Gerontology, University of Southern California, Los Angeles, CA 90089

**Keywords:** Autophagy, Proteostasis, *C. elegans*, Aging, Protein aggregation, Inter-tissue signaling

## Abstract

Proteostasis (protein homeostasis), the balance of protein synthesis, folding, and degradation, is critical for cellular function and organismal health. Its disruption leads to the accumulation of misfolded and aggregated proteins, hallmarks of aging and age-related diseases, including neurodegeneration. Autophagy, a conserved lysosome-mediated degradation pathway, is central to proteostasis by clearing toxic proteins and damaged organelles. In *Caenorhabditis elegans*, studies across conserved longevity paradigms and models of neurodegenerative diseases have defined key mechanisms by which autophagy maintains proteostasis during aging and stress. Beyond its degradative functions, autophagy contributes to spatial quality control by promoting the formation of potentially protective protein inclusions and coordinating with the ubiquitin-proteasome system. Emerging evidence also points to noncanonical autophagy pathways, such as unconventional secretion and inter-tissue communication, that broaden its role in systemic proteostasis. Together, these advances underscore autophagy’s multifaceted contribution to protein quality control, with wide-ranging implications for aging, stress resistance, and neurodegenerative disease.

## Introduction

Cells maintain protein homeostasis, or proteostasis, through a conserved quality control system called the proteostatic network, which regulates protein synthesis, folding, trafficking, maintenance, and degradation. The proteostatic network includes molecular chaperones, the ubiquitin-proteasome system (UPS), and autophagy.^1^ Molecular chaperones play a key role in protein folding and preventing aggregation by recognizing, stabilizing, and refolding misfolded proteins. If damaged or misfolded proteins cannot be refolded, they are targeted for degradation by the UPS or autophagy.^2^ Since the disruption of proteostasis is a hallmark of aging and many age-related diseases,^3^ understanding the molecular mechanisms that regulate proteostasis may provide insight into proteinopathies, including neurodegenerative disorders such as Alzheimer’s diseases (AD), Huntington’s disease (HD), Parkinson’s disease (PD), and amyotrophic lateral sclerosis (ALS).

Many of the key discoveries in autophagy regulation and its role in proteostasis have been made in genetically tractable model organisms. In particular, the nematode *Caenorhabditis elegans* has emerged as a powerful and enduring model system due to its conserved proteostasis network, short lifespan, transparency for *in vivo* imaging, multicellular complexity, and versatile tools for tissue-specific genetic manipulation. For example, tissue-specific fluorescent reporters allow dynamic tracking of autophagy and protein aggregation *in vivo*, and RNAi (RNA interference) strategies, as well as degron-based approaches, allow precise dissection of proteostasis mechanisms at both the tissue and organismal level. Importantly, *C. elegans* remains uniquely valuable for uncovering conserved principles of proteostasis because of its experimental tractability and unparalleled ability to link cellular pathways to organismal physiology. This review focuses on the role of autophagy in proteostasis, emphasizing findings from *C. elegans* to illustrate how autophagy maintains protein quality control at the molecular, tissue, and whole-organism levels. We explore its roles in degradation, aggregation compartmentalization, stress responses, and inter-tissue signaling across both physiological and disease contexts.

## Molecular mechanisms of autophagy in proteostasis

Autophagy is broadly classified into three subtypes: macroautophagy, microautophagy, and chaperone-mediated autophagy (CMA).^4^ Among these, macroautophagy is the only form well characterized in *C. elegans* and is the focus of this review. Macroautophagy is distinguished by the formation of a double-membrane vesicle called the autophagosome, which engulfs cytosolic cargo, including damaged or aggregated proteins, organelles, or pathogens.^4^ Autophagosomes, once fully closed around the cargo, fuse with lysosomes, wherein hydrolases break down the cargo into basic components for recycling. In contrast, microautophagy involves the direct invagination or protrusion of the lysosomal membrane to engulf cytosolic material without the need for an intermediary vesicle. CMA is even more selective and involves recognition of substrate proteins bearing a KFERQ (pentapeptide sequence Lys-Phe-Glu-Arg-Gln)-like motif, which are delivered individually to the lysosomal membrane by chaperone complexes, such as HSC70, and translocated across the membrane via the CMA receptor isoform of lysosome-associated membrane protein 2, LAMP2A.^4^, ^5^ While many proteins in *C. elegans* contain a KFERQ motif, there is no homolog for LAMP2A, and it is not clear whether *C. elegans* proteins can be directly delivered to lysosomes for degradation by CMA or microautophagy.^6^ It therefore remains unclear whether such mechanisms can contribute to proteostasis in *C. elegans.*

**Fig. 1:**
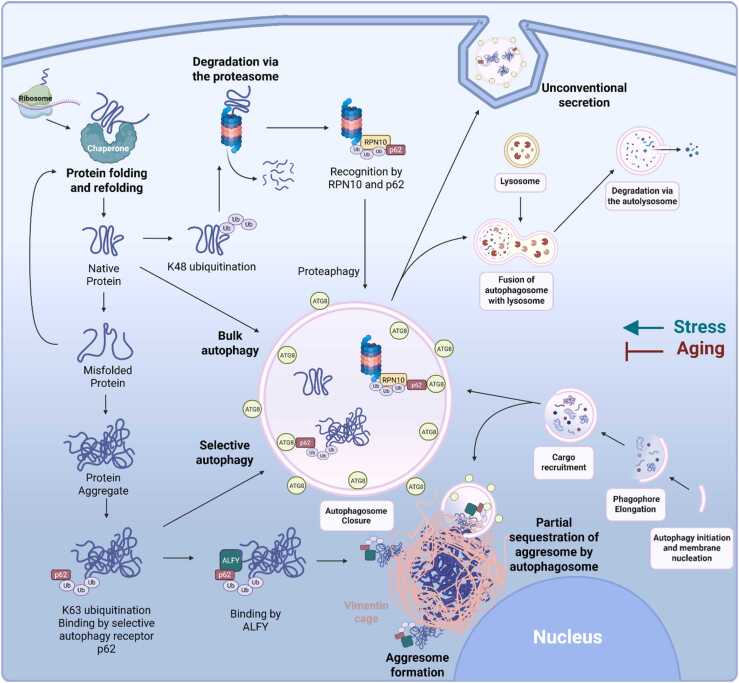
*The role of autophagy in proteostasis.* Newly synthesized proteins undergo folding and refolding with the help of molecular chaperones. Misfolded or damaged proteins are recognized and tagged with ubiquitin chains for degradation. The proteasome removes short-lived or soluble misfolded proteins tagged with K48-linked polyubiquitin chains. Proteasomes can be degraded by autophagy via “proteaphagy” and bulk and selective autophagy pathways degrade larger protein aggregates via specific cargo receptors including p62/SQSTM1 that recognize K63-linked polyubiquitin chains. If not degraded, p62/SQSTM1-bound aggregates may be recognized by autophagy-linked FYVE protein ALFY and surrounded by a vimentin cage near the nucleus. Aggresomes may be partially sequestered by autophagosomes. Protein aggregates can be exported through unconventional secretion pathways, which may involve autophagy machinery proteins. Autophagy declines with age, while stress conditions activate autophagy to help maintain proteostasis. Figure created in BioRender (https://www.biorender.com/).

Macroautophagy (hereafter referred to as autophagy) is an evolutionarily conserved process first defined in yeast, with most core molecular components shared across eukaryotes. Here, we use yeast and human nomenclature for general descriptions and reference *C. elegans* orthologs where appropriate. Autophagy is a tightly regulated, multi-step process requiring at least 40 autophagy-related (*ATG*) genes (Figure 1). It proceeds through distinct stages: initiation, phagophore formation, expansion, membrane closure, transport to and fusion with the lysosome, and degradation.^7^ At initiation, phosphorylation of the Atg1/ULK1 (UNC-51 in *C. elegans*) kinase complex triggers the recruitment of the class III phosphatidylinositol 3-kinase complex, resulting in synthesis of phosphatidylinositol 3-phosphate lipids required for the nucleation of the phagophore.^8^ Membrane expansion depends on lipid delivery by Atg9 and the conjugation of Atg8 family proteins (LGG-1/GABARAP and LGG-2/LC3 in *C. elegans*) to both the inner and outer membrane. This begins with Atg4, a protease that cleaves Atg8 proteins, exposing a C-terminal glycine residue. This exposed glycine allows Atg8 to be conjugated to phosphatidylethanolamine by the Atg12–Atg5–Atg16 complex, anchoring Atg8 to the growing autophagosomal membrane.^9^ Atg8 proteins on the inner autophagosomal membrane facilitate selective recruitment of cargo to the membrane, while outer Atg8s facilitate transport and fusion with the lysosome.^10^ Autophagosome fusion with the lysosome (or vacuole in yeast) requires coordinated action of membrane tethering and SNARE machinery. The conserved HOPS complex facilitates this process by promoting SNARE-dependent membrane fusion. Although the HOPS complex is conserved in *C. elegans*, the specific SNARE proteins involved in autophagosome–lysosome fusion are less well defined. After formation of the autolysosome, cargo is degraded by lysosomal hydrolases^11^ and the breakdown products, such as amino acids and fatty acids, are recycled back into cellular metabolism.^4^

In addition to the core machinery, autophagy is regulated by transcriptional programs. In *C. elegans*, the transcription factor HLH-30/TFEB broadly induces autophagy and lysosome genes, DAF-16/FOXO, PHA-4/FOXA, SKN-1/NRF2, and HSF-1 activate overlapping subsets under nutrient deprivation and stress conditions.^12^, ^13^ Nutrient- and energy-sensing pathways (mTORC1, insulin/IGF, and AMPK) converge on these factors to coordinate autophagosome biogenesis.^14^

## Autophagy and UPS: core functions and crosstalk

Autophagy and the UPS are distinct arms of the proteostasis network that function in parallel to maintain protein quality control (Figure 1). While they primarily handle different substrates, both systems respond to proteotoxic stress and exhibit regulatory crosstalk. The UPS primarily degrades short-lived, misfolded, or regulatory proteins that are marked for degradation by polyubiquitin chains and processed by the 26S proteasome. These proteins are unfolded, translocated into the proteasome core, and cleaved into peptides for recycling.^15^ In mammalian cells, K48-linked polyubiquitin chains serve as the canonical signal for proteasomal degradation, while K63-linked chains and monoubiquitination are more commonly associated with autophagic degradation.^16^ Although similar linkages are assumed in *C. elegans*,^17^ direct evidence for linkage-specific sorting in this model is limited.

While it is commonly assumed that impairment of one degradative system leads to compensatory upregulation of the other, experimental evidence indicates that this is not universal. An instance of compensation is seen upon depletion of disaggregating chaperones in *C. elegans,* which impairs UPS capacity but induces autophagy.^18^ In contrast, expression of amyloidogenic proteins such as Aβ in neurons or polyQ40 in muscle impairs both autophagy and the UPS, even in tissues where toxic proteins are not expressed, indicating that proteotoxic stress can simultaneously compromise both systems and propagate across tissues.^18^ Proteasome function can also be disrupted by RNAi against the proteasome-associated deubiquitinating enzymes (DUBs) *ubh-4*/*UCHL5* or *usp-14/USP14*.^19^ Unlike most DUBs that act on selective substrates, these enzymes are physically integrated into the proteasome, where they deubiquitinate substrates as they are engaged by the proteasome for degradation, thereby controlling both substrate processing and ubiquitin recycling. Their inhibition reduces autophagic flux in mammalian cells and triggers tissue-specific effects in *C. elegans.* In the intestine, *ubh-4* or *usp-14* knockdown decreases autophagosome abundance without affecting autolysosomes, while other tissues such as seam cells and the pharynx show distinct responses.^19^ Although the underlying mechanisms remain unresolved, these findings underscore that proteasome-associated DUBs modulate autophagy in a tissue-dependent manner.

Autophagy can also target proteasomes for degradation, termed “proteophagy,” highlighting their bidirectional relationship.^20^ This has been observed in yeast and mammalian systems but not yet directly demonstrated in *C. elegans*.^21^, ^22^, ^23^ Conversely, certain autophagy-related proteins, such as beclin, BECN1, can be degraded by the proteasome in specific contexts, such as mTORC1 activation (which suppresses autophagy under nutrient-rich conditions), caspase cleavage (which occurs during apoptosis), or oxidative stress (which damages cellular components and can modulate degradation pathways).^24^ Collectively, these findings indicate that these systems are not interchangeable safeguards but interdependent regulators whose disruption can destabilize the proteostatic network. Thus, rather than viewing their relationship as compensatory, future work in *C. elegans* should treat autophagy—UPS crosstalk as a central determinant of organismal proteostasis and longevity. While many aspects of their interaction have been characterized in mammalian systems,^25^ further studies in *C. elegans* are needed to determine how these two degradative pathways interplay during development, aging, and stress.

## Selective autophagy: aggrephagy

Autophagy can be either nonselective (bulk) or selective.^26^ In selective autophagy, cargo-specific receptors guide the delivery of particular substrates to the forming autophagosome (Figure 1). For example, p62/SQSTM1 (SQST-1 in *C. elegans*) is a well-characterized selective autophagy receptor that binds to ubiquitinated protein aggregates and simultaneously interacts with Atg8 proteins (LC3/GABARAP in mammals) on the phagophore membrane through its LC3-interacting region. This interaction facilitates the targeted engulfment of protein aggregates into autophagosomes in a process termed aggrephagy.^27^ In *C. elegans,* SQST-1/p62 overexpression alleviates paralysis in mutants expressing temperature-sensitive (ts) misfolding proteins (neuronal *dyn-1(ts)*, intestinal *let-60(ts)*, and muscle *unc-54(ts)*),^28^ likely due to increased degradation of aggregates, since this rescue is partially dependent on *lgg-1/ATG8/GABARAP*. Moreover, SQST-1/p62 overexpression extends lifespan in an autophagy-gene dependent manner, requiring core autophagy genes *bec-1/ATG6/BECN1*, *lgg-1/ATG8/GABARAP*, *vps-34*, *atg-18*, *epg-5*, and *cup-5/MCOLN1*, and is associated with increased autophagosome formation in neurons and intestinal cells.^28^ The ability of SQST-1/p62 to promote autophagosome formation has also been observed in mammalian systems, where p62 can localize to autophagosome formation sites prior to LC3/Atg8 recruitment, and where ubiquitinated cargo can stimulate phase separation and membrane remodeling in human and murine cell lines.^29^, ^30^, ^31^ These findings raise the possibility that SQST-1/p62 itself may contribute to autophagy initiation by nucleating the autophagy machinery at sites of cargo accumulation. While such condensates promote cargo clustering and delivery to autophagosomes, excessive or immobilized SQST-1/p62 on aggregates can block the recruitment of other selective autophagy receptors, thereby hindering cooperative degradation pathways and ultimately compromising proteostasis.^29^, ^32^ This illustrates that the contribution of SQST-1/p62 to proteostasis is not uniformly beneficial. Its overexpression at 25 °C shortens *C. elegans* lifespan, coinciding with its progressive accumulation and increased polyubiquitinated protein levels.^33^ Consistent with this, expansion of lipid droplet stores either through *daf-2* mutation or through inactivation of the lipid droplet-associated lipase *atgl-1/PNPLA2* in wild-type animals reduces SQST-1/p62 accumulation and extends lifespan,^33^ thereby linking lipid metabolism to the regulation of SQST-1/p62 dynamics and proteostasis. These findings highlight the context-dependent nature of SQST-1/p62: while it can promote aggregate clearance, reinforce the proteostasis network, and extend lifespan, its excessive accumulation or restricted dynamics may interfere with other degradative pathways and exert detrimental effects under specific thermal or disease conditions.

## Age-related changes in proteostasis in *C. elegans*

Aging in *C. elegans* is accompanied by a progressive decline in proteostasis, marked by increased accumulation of misfolded and aggregation-prone proteins. This decline occurs across multiple tissues and is also demonstrated in disease models where protein aggregation is exacerbated with age. Proteome-wide analyses show that hundreds of endogenous proteins in *C. elegans*, including the aggregation-prone GTPase RHO-1 and casein kinase KIN-19, become increasingly insoluble and form amyloid-like deposits with age.^34^ Complementing these observations, quantitative mass spectrometry identified SDS-insoluble proteins that markedly increase in aged animals.^35^ More than 200 proteins are enriched in the insoluble fraction, with strong over-representation of ribosomal/translation and mitochondrial energy functions. RNAi knockdown of ∼41% of these “insoluble-set” genes extends lifespan, directly linking age-dependent protein insolubility to longevity regulation. Consistent with these findings, a large-scale, quantitative proteomic study of >5000 proteins showed that about one-third of these proteins change in abundance by at least 2-fold, either increasing or decreasing during aging, leading to severe proteome imbalance.^36^ These changes in protein expression are accompanied by widespread protein aggregation, particularly among highly expressed proteins dominating aggregate load. Despite these changes, proteasome subunits and activity increase with age in *C. elegans*, in contrast to yeast, flies, and mammals, where proteasome function declines.^37^, ^38^, ^39^, ^40^, ^41^, ^42^, ^43^, ^24^ Targeted molecular analyses focused on the key regulatory proteasomal subunit RPN-6.1/PSMD11, critical for maintaining proteasome function and lifespan, suggest age-related decline and a role for age-related proteasome impairment in longevity regulation,^44^, ^45^ in line with other models. Reconciling these findings may require considering tissue-specific expression, post-translational regulation, and the distinction between proteasome quantity and functionality.

In addition to these proteome-wide studies, ubiquitinome profiling in *C. elegans* revealed extensive age-dependent changes in protein ubiquitination.^46^ Across ∼1000 proteins, both losses and gains of ubiquitination sites were observed, with a net overall decline in ubiquitination, leading to the conclusion that aging is accompanied by a global decline in ubiquitination.^46^ Mechanistically, this loss is driven by an increase in the activity of DUBs, since pharmacological or genetic inhibition of DUBs restores ubiquitination in old worms and reduces the accumulation of specific proteasome substrates. Studies in the brains of vertebrate models, mice and killifish, show that aging is also associated with a complex rewiring of the ubiquitinome, with many sites showing increased ubiquitination and others a decrease, underscoring species- and tissue-specific differences in ubiquitin dynamics.^47^

In *C. elegans*, aging is similarly associated with selective remodeling at the transcriptional level. Early microarray studies highlighted that only ∼1% of genes change consistently with age, with strong enrichment for stress-response genes such as HSPs.^48^, ^49^ More recent transcriptomic work extends this view, revealing “transcriptional drift,” a loss of coordinated gene regulation that produces both transcriptional increases and decreases, reminiscent of proteome and ubiquitinome remodeling.^50^ This transcriptional drift in *C. elegans* particularly affects stress-responsive pathways that overlap with proteostasis, including chaperones, detoxification systems, and immune regulators. A similar age-related drift has been observed in human fibroblasts, where cells lose coordinated expression of stress response and protein quality control genes, further weakening the stress resilience network.^51^

In addition to whole-body proteomic and transcriptional studies, age-related proteostasis alteration can be tissue-specific. For example, the aggregation-prone proteins RHO-1 and KIN-19 accumulate specifically in the pharynx during aging. Intriguingly, genetic inhibition of key protein quality control pathways, including chaperones (*hsf-1*, *hsp-1/SSA1–4/HSPA8*, *dnaj-1/YDJ1/DNAJA1*), the UPS (*rpn-6.1/PSMD11*, *pas-6/PRE1/PSMA1*), and macroautophagy (*lgg-1/ATG8/GABARAP*, *bec-1/ATG6/BECN1*, *atg-18*, *unc-51/ATG1/ULK1*), reduces pharyngeal aggregation.^52^ Notably, loss of *vha-12* or *scav-3*, which regulate lysosomal acidification and membrane integrity, respectively, abolish this protective effect and restore RHO-1 aggregation, demonstrating that lysosomal degradation is required. Together, these findings indicate that a lysosome-dependent “safety mechanism” eliminates protein aggregates when canonical macroautophagy or UPS function is compromised. A similar pattern is observed in the germline. RHO-1, along with NMY-2 (non-muscle myosin) and HCP-1 (centromere protein), also forms visible aggregates within oocytes during aging.^53^ These proteins, which are normally cleared efficiently, form visible aggregates with age, suggesting an age-associated decline in clearance mechanisms. While lysosomal activity remains active in the germline and contributes to aggregate clearance, inhibition of autophagy genes such as *bec-1/ATG6/BECN1* or *atg-18* does not exacerbate aggregation.^53^ The persistence of lysosome-dependent clearance despite loss of macroautophagy genes points toward alternative pathways. Microautophagy, which involves the direct engulfment of cytosolic material by lysosomal invagination, represents a plausible mechanism, as it could account for lysosome-dependent but macroautophagy-independent degradation.

## Age-related changes in autophagy in *C. elegans*

Autophagy in *C. elegans* is also affected by age with the number of autophagic vesicles increasing with age.^54^, ^55^ Spatiotemporal flux assays using tandem-tagged LGG-1/Atg8/GABARAP reporters and Bafilomycin A, an inhibitor of lysosomal acidification, revealed that this increase in autophagosome number reflects stalled autophagy rather than an induction.^55^ This study showed that autophagic activity declines in the intestine, muscle, pharynx, and neurons of aging wild-type animals, with defects emerging after autophagosome formation.^55^ In parallel, the actin cytoskeleton undergoes age-dependent disorganization in *C. elegans* tissues, including muscle, intestine, and hypodermis.^56^ Disruption of actin dynamics impairs multiple stages of the autophagy pathway, including autophagosome formation, maturation, and trafficking.^57^, ^58^ Actin also facilitates proteasomal degradation by trafficking ubiquitinated cargo.^59^ Thus, cytoskeletal collapse may compromise both autophagy and UPS function, contributing to proteostatic decline during aging. Age-dependent changes in lysosomal morphology, acidity, motility, and degradation capacity further underscore the functional decline of autophagy, which is accompanied by the age-related transcriptional decrease of 43 lysosome-related genes, including key V-ATPase subunits and cathepsin proteases, supporting a broad reduction in lysosomal competence.^60^ Recent single-cell RNA-seq studies demonstrate that aging is accompanied by selective and tissue-specific transcriptional changes in *C. elegans,* where select autophagy-related genes are upregulated with age, including transcription factor *hlh-30/TFEB*, the master regulator of autophagy- and lysosomal-related genes.^61^, ^62^ Consistent with these findings, conserved longevity pathways have transcriptionally upregulated autophagy genes to preserve healthspan and lifespan.^12^, ^60^, ^63^ Proteomic analysis also revealed age-related changes in expression of autophagy proteins, most prominently the strong upregulation of the selective autophagy receptor SQST-1/p62, consistent with impaired autophagic flux.^36^ Other autophagy-related proteins, like LGG-1/Atg8/GABARAP and ATG-7 are also increased, while ATG-18 and certain V-ATPase subunits are reduced with age. Further data analysis will be needed to fully resolve the age- and cell-type-specific transcriptional and proteomic changes in autophagy. Nevertheless, upregulation of autophagy genes and proteins in specific tissues with age may reflect a compensatory response to proteostatic stress rather than increased autophagy flux or efficacy. Collectively, these findings indicate that autophagy undergoes age-related changes that are tissue-specific and likely contribute to differential proteostasis failure across cell types, highlighting the need to consider spatial regulation when evaluating the role of autophagy in aging.

## Autophagy and protein aggregation models in *C. elegans*

**Fig. 2:**
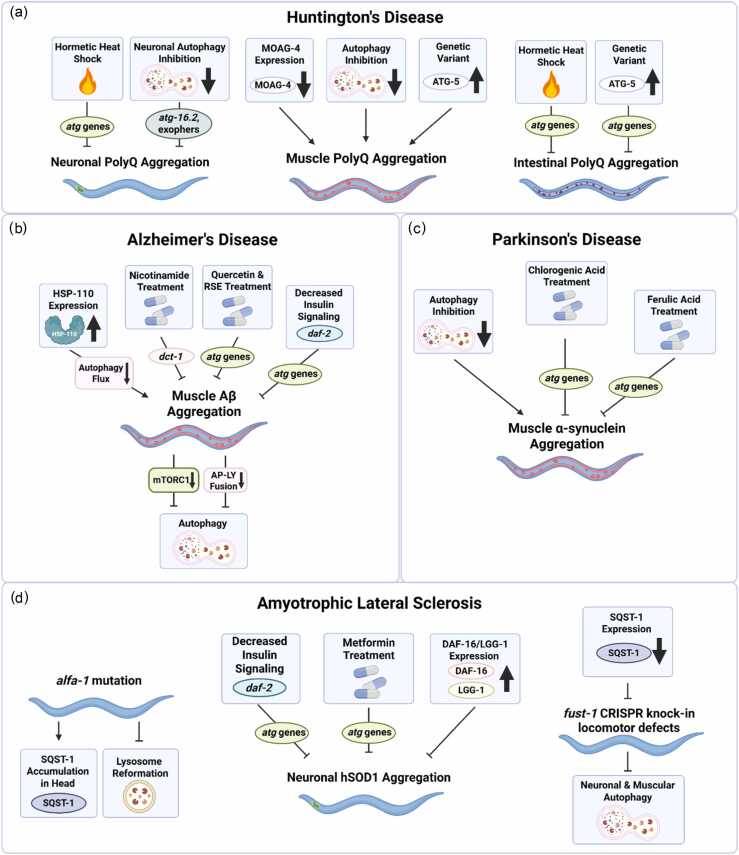
*Autophagy affects protein aggregation in C. elegans neurodegenerative disease models.* (**a**) In *C. elegans* models of Huntington’s disease, expanded polyglutamine (polyQ) aggregation can be decreased by hormetic heat shock in an autophagy (*atg*) gene-dependent manner in neurons (left) and intestine (right). Reduced *moag-4/SERF* expression decreases polyQ aggregation in muscle (middle). Autophagy induction via increased ATG-5 levels, caused by a natural *atg-5* variant increases PolyQ aggregation in the muscle (middle) but decreases polyQ aggregation in the intestine (right). (**b**) In *C. elegans* Alzheimer’s disease models, *hsp-110* overexpression impairs autophagic flux and incresaes Aβ aggregation in the muscle. Treatment with nicotinamide, quercetin, and Radix Stellariae Extract (RSE) improves Aβ phenotypes in a *dct-1*- and *atg* gene-dependent manner. Expression of Aβ in muscle leads to increased mTORC1 activation and blocked autolysosomal (AP-LY) fusion, leading to decreased autophagy flux in *C. elegans* expressing muscle Aβ. (**c**) In *C. elegans* Parkinson’s disease models, α-synuclein aggregation in the muscle is increased by inhibition of *atg* genes, while chlorogenic acid and ferulic acid treatments decrease aggregation in an *atg*-dependent manner. (**d**) In *C. elegans* models of Amyotrophic Lateral Sclerosis, mutations in *alfa-1* lead to SQST-1 accumulation in the head and lysosome reformation defects (left). Decreased insulin signaling (*daf-2*) and metformin treatment reduce neuronal hSOD1 aggregation in an autophagy-dependent manner. *daf-16/FOXO* and *lgg-1/Atg8/GABARAP* overexpression reduces hSOD1 aggregation (middle). In *fust-1* CRISPR knock-in animals, neuronal and muscular autophagy is reduced; however, loss of *sqst-1/p62* suppresses *fust-1* locomotor defects (right). Figure created in BioRender (https://www.biorender.com/).

*C. elegans* offers a powerful system for modeling age-associated proteostasis decline through both endogenous and disease-relevant aggregation paradigms. Endogenous models include strains with ts missense mutations in proteins that misfold at restrictive temperatures.^64^ In animals carrying ts mutations in body-wall muscle genes *unc-15* and *unc-54*, RNAi-knockdown of core autophagy genes *unc-51/ATG1/ULK1* and *bec-1/ATG6/BECN1* significantly worsens paralysis, indicating that autophagy is essential for maintaining proteostasis in these conditions.^65^ In addition, endogenous aggregation-prone proteins such as RHO-1, NMY-2, and HCP-1 form visible GFP-tagged aggregates in germ cells with age, revealing a tissue-specific decline in proteostasis within the germline.^53^ However, the role autophagy plays in managing these aggregates remains unknown. In parallel, *C. elegans* disease-relevant models expressing aggregation-prone proteins linked to neurodegenerative diseases, including models of HD,^66^, ^67^ AD,^68^ PD,^69^ and ALS^70^ have expanded our understanding of autophagy’s role in these proteinopathies (Figure 2).

### Autophagy in *C. elegans* HD models

In humans, HD is caused by the expansion of polyglutamine (polyQ) repeats in the *Huntingtin* (*HTT*) gene, leading to misfolding and aggregation of the HTT protein, which disrupts proteostasis and contributes to neurodegeneration. *C. elegans* models expressing polyQ expansions in specific tissues recapitulate neuronal dysfunction and paralysis and allow for visualization of fluorescently tagged polyQ proteins to assess aggregation^66^, ^67^, ^71^ (Figure 2a). These models have enabled discovery of proteostasis modulators, including MOAG-4/SERF, which promotes polyQ aggregation in *C. elegans* muscle by binding to amyloidogenic proteins, disrupting protective intramolecular interactions, and accelerating conversion into aggregation-prone conformations. MOAG-4/SERF is thus a candidate therapeutic target for blocking early nucleation steps in protein misfolding diseases like HD and PD.^72^ Autophagy is critical for proteostasis in the muscle, where loss of autophagy genes, including *unc-51/ATG1/ULK1, bec-1/ATG6/BECN1*, *atg-7,* and *atg-18*, increases polyQ aggregation.^65^, ^73^ While autophagy impairment accelerates polyQ aggregation and associated pathology, whether autophagy induction clears aggregates remains an open question. However, hormetic heat shock, which increases lifespan and stress resistance in *C. elegans* and induces autophagy, also reduces polyQ aggregation in neurons and intestine in an autophagy- and *sqst-1/p62-*dependent manner. Interestingly, hormetic heat shock has no effect in the muscle, highlighting tissue-specific differences in the proteostatic response to heat stress.^28^, ^65^ Further highlighting this complexity, expression of a natural genetic *atg-5* variant, which elevates ATG-5 abundance and increases autophagy, reduced intestinal polyQ aggregation but unexpectedly increased polyQ aggregation in the body-wall muscle.^74^ Together, these findings indicate that autophagy limits polyQ aggregation and toxicity in *C. elegans*. While tissue-specific differences indicate that autophagy induction may not act uniformly across cell types, the consistent requirement for autophagy genes in protecting against proteotoxicity strongly supports autophagy enhancement as a viable therapeutic strategy in HD.

### Autophagy in *C. elegans* AD models

The aggregation of β-amyloid (Aβ) peptides is a key feature of AD and linked to proteostasis disruption and neurodegeneration. Mounting evidence suggests that impaired autophagic function in AD accelerates disease advancement.^75^ In *C. elegans* AD models, muscle-specific Aβ-expression activates mTORC1 signaling, which inhibits autophagy by preventing the nuclear translocation of HLH-30/TFEB, decreasing the transcription of autophagy and lysosomal genes.^76^ Autophagosomal structures accumulate in these animals, resembling observations in AD patient brains,^77^ and indicating a defect in late-stage autophagy, where autophagosomes fail to fuse with lysosomes for degradation.^77^ Although muscle Aβ expression increases lysosomal gene expression and lysosomal acidity, suggesting that lysosomes remain functional, autophagy flux remains defective^77^ (Figure 2b). Following Aβ induction, the levels of reactive oxygen species increase and fusion-related genes are downregulated, suggesting that Aβ-induced reactive oxygen species interfere with autophagosome-lysosome fusion.^77^ Pharmacological inhibition of autophagosome formation using 3-methyladenine improves neuronal function and delays paralysis in Aβ-expressing *C. elegans*, likely by reducing autophagosome accumulation when degradation is impaired. Under these conditions, compensatory proteostasis mechanisms are induced, particularly small molecular chaperones, including HSP-16.2 are upregulated to relieve intracellular stress.^77^ Knockdown of chaperone genes reverses the benefit of 3-methyladenine-mediated autophagy suppression on paralysis in Aβ-expressing animals, underscoring their role in maintaining protein quality control under impaired autophagic flux in *C. elegans* AD models.^77^

While these studies illuminate how Aβ aggregation disrupts downstream proteostasis, upstream regulators also influence the aggregation process itself. Unexpectedly, overexpression of the chaperone gene *hsp-110/SSE1/HSPH1* in *C. elegans* impairs autophagic flux and leads to increased Aβ aggregation, while *hsp-110* depletion restores autophagic clearance and reduces aggregation in neurons^78^ (Figure 2b). These findings suggest that dysregulated chaperone activity may contribute to autophagy failure in AD, highlighting a previously underappreciated mechanism by which proteostasis network imbalance drives disease pathology. However, this effect contrasts with findings in *daf-2* mutants, where reduced insulin signaling alleviates Aβ aggregation and paralysis in an autophagy-dependent manner,^79^ highlighting the context-dependent role of autophagy in regulating protein aggregation in *C. elegans* AD models.

Several studies have evaluated pharmacological autophagy modulators for their potential to mitigate Aβ-induced proteotoxicity in *C. elegans*. Compounds such as quercetin and Radix Stellariae Extract improve proteostasis and reduce disease phenotypes in *C. elegans,* supporting the potential of therapeutic autophagy enhancement.^80^, ^81^

Mitochondrial quality control through selective mitophagy also plays a protective role in AD. In *C. elegans*, treatments that either boost NAD^+^ levels (e.g., nicotinamide riboside) or induce mitochondrial stress (e.g., inhibition of mitochondrial translation) activate mitophagy and reduce Aβ aggregation and toxicity,^82^ and require the mitophagy receptor DCT-1/BNIP3 for these protective effects. Corroborating these findings, studies in human AD cortex and transgenic AD mice reveal elevated transcripts of mitophagy factors, including *p62*, *PINK1, LC3, DNM1L*, and *BECN1.*^82^ This transcriptional signature suggests that upregulation of mitochondrial clearance mechanisms may be a conserved response to proteotoxic stress in AD.

Autophagy acts as a context-dependent regulator of Aβ toxicity in *C. elegans* AD models. Impaired flux accelerates proteostasis collapse, while targeted modulation, via chaperones, pharmacological enhancers, or mitophagy, restores clearance and alleviates disease phenotypes. Autophagy therefore functions as a critical regulator of AD progression and remains a promising therapeutic target.

### Autophagy in *C. elegans* PD disease models

Patients diagnosed with PD experience impaired motor function, including reduced balance and tremors, along with non-motor symptoms of depression, anxiety, dementia, and sleep disruption.^69^ These symptoms typically present later in life and are primarily associated with the progressive loss of dopaminergic neurons and the aggregation of alpha-synuclein into Lewy bodies.^83^ Autophagy dysfunction contributes to α-synuclein accumulation and may play a central role in disease progression.^84^ PD can be modeled in *C. elegans* using loss-of-function mutations in *pink-1*, *pdr-1/PRKN,* or through transgenic expression of fluorescently tagged α-synuclein.^69^ Chlorogenic acid and ferulic acid, polyphenols found in coffee and tea, have shown neuroprotective effects in preclinical models, including *C. elegans* and zebrafish, although they are not approved PD therapies in humans.^85^, ^86^, ^87^ Chlorogenic acid reduces α-synuclein aggregation in muscle, preserves dopaminergic neurons, and improves motor function. These effects correlate with increased LGG-1/Atg8 puncta and upregulation of autophagy genes *bec-1/ATG6/BECN1, lgg-1/ATG8/GABARAP, unc-51/ATG1/ULK1,* and *vps-34* after treatment^85^ (Figure 2c). Similarly, ferulic acid reduces muscular α-synuclein aggregation and improves motor function, accompanied by increased mRNA levels of *lgg-1/ATG8/GABARAP, vps-34,* and *unc-51/ATG1/ULK1.* Knockdown of these genes blocks the protective effects, confirming their requirement for reduced aggregation.^86^ Supporting this mechanism, oolong tea extract has also been shown to reduce age-related protein aggregation in *C. elegans* muscle via polyphenols, further linking these compounds to enhancement of proteostasis potentially through autophagic clearance.^88^ These findings underscore the potential of autophagy as a therapeutic target in the treatment of PD and highlight the need for further investigation into the role that autophagy plays in additional models of PD.

### Autophagy in *C. elegans* ALS models

ALS is a fatal neurodegenerative disease marked by progressive motor neuron loss, muscle denervation, and protein aggregation.^89^ While most ALS cases are sporadic, mutations in more than 45 human genes, including *sod-1/SOD1*, *TARDBP* (encoding *TDP-43*), *FUS*, and *alfa-1/C9orf72* have been identified as genetic drivers.^90^ *C. elegans* ALS models are generated by expressing these human proteins or their *C. elegans* homologs in neurons or muscle, where they form aggregates and cause locomotor defects, thereby recapitulating aspects of ALS pathology.^70^ *C. elegans* expressing mutant *hSOD1* develop motor dysfunction, which is suppressed in long-lived *daf-2* mutants, potentially through enhanced autophagy, although direct tests of autophagy gene requirements in this context remain outstanding.^91^ Supporting a protective role for autophagy, pharmacological activation with metformin improves locomotion, extends lifespan, and reduces hSOD1 aggregation dependent on *daf-16/FOXO* and *lgg-1/ATG8/GABARAP*, while overexpression of either gene alone is sufficient to ameliorate ALS-associated phenotypes^92^ (Figure 2d). Consistently, CRISPR knock-in models of *fust-1/FUS* reveal that ALS-linked mutations disrupt neuronal and muscular autophagy, leading to stress-induced neuromuscular dysfunction and accumulation of SQST-1/p62. Notably, loss of *sqst-1/p62* suppresses locomotion and neuromuscular defects but does not restore autophagy flux, indicating that impaired autophagy lies upstream of SQST-1/p62 toxicity in ALS pathogenesis.^93^ Interestingly, ALS phenotypes are not dictated by cell-autonomous aggregation alone but are influenced by systemic proteostasis regulation. For example, germline-to-soma signaling can trigger mitochondrial stress and drive FUS and TDP-43 aggregation.^94^

*C. elegans* expressing expanded hexanucleotide repeats associated with human C9orf72 exhibit paralysis, reduced lifespan, and additional motor deficits due to the synthesis of toxic dipeptide repeats by repeat-associated non-AUG translation.^95^ Deletion of the *C. elegans’* C9orf72 orthologue *alfa-1* also models ALS, resulting in motor neuron degeneration and increased sensitivity to stress.^96^ Loss of *alfa-1* reduces the number and length of lysosomal tubular structures in embryos, which normally emerge during lysosomal reformation following degradation, suggesting that *alfa-1* mutants have impaired lysosomal function. Further supporting a link with autophagy, in adulthood *alfa-1* mutants accumulate SQST-1/p62 in the head region, indicative of defective cargo clearance, although direct flux experiments are needed to confirm this block^97^ (Figure 2d). By contrast, in murine models, loss of C9orf72 dysregulates mTORC1 signaling, leading to enhanced TFEB expression and elevated autophagy flux.^98^ Thus, C9orf72 dysfunction can either impair or hyperactivate autophagy depending on species and cellular context. Nevertheless, these studies argue that insufficient autophagy is a driver of ALS-like pathology in *C. elegans* and that genetically or pharmacologically boosting autophagy is a plausible, testable therapeutic strategy, with the important caveat that efficacy is tissue-context dependent.

## Autophagy and proteostasis in *C. elegans* longevity paradigms

**Fig. 3:**
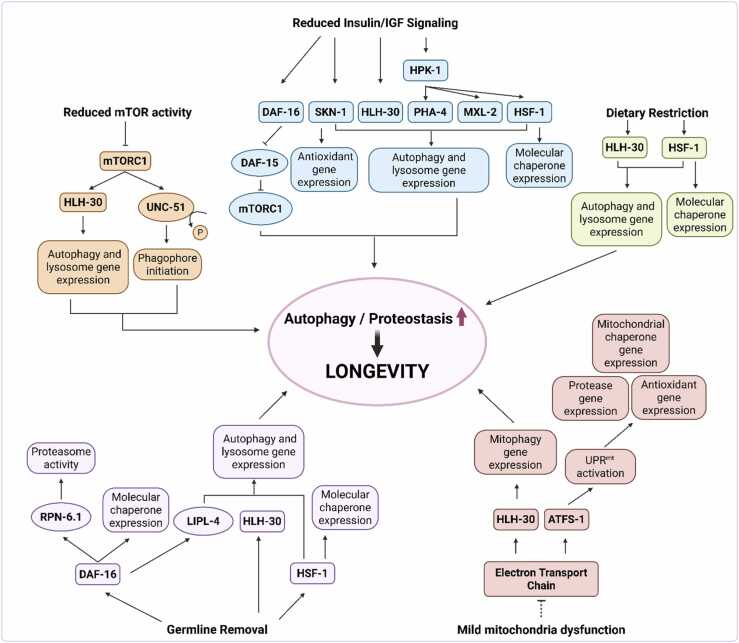
*Conserved longevity pathways converge on autophagy and proteostasis to extend lifespan.* Long-lived *C. elegans* models, including those with reduced insulin/IGF-1 signaling, reduced mTOR activity, dietary restriction, germline removal, or mild mitochondrial dysfunction, exhibit enhanced autophagy and improved proteostasis and activate additional autophagy-independent mechanisms (not shown) that contribute to lifespan extension. These paradigms activate overlapping networks of transcription factors that regulate autophagy, lysosomal function, chaperones, antioxidant responses, and proteasome activity. Autophagy serves as a central integrator of these signals, contributing to the maintenance of protein homeostasis and promoting organismal longevity. Figure created in BioRender (https://www.biorender.com/).

Autophagy acts as a critical effector in multiple conserved longevity paradigms, including reduced insulin/IGF-1 signaling (IIS), reduced mTORC1 signaling, dietary restriction (DR), germline-less animals (*glp-1*), and mild mitochondrial dysfunction, all of which delay aging by promoting proteostasis (Figure 3). Specifically, these long-lived *C. elegans* models exhibit sustained or enhanced autophagic activity, which is required for lifespan extension and linked to improved proteostasis. Many longevity paradigms also remodel lipid metabolism in ways that intersect with autophagy: some *daf-2* and germline-less mutants accumulate lipid droplets, whereas DR reduces lipid stores.^99^ Both expansion and depletion of lipid pools can be linked to lifespan extension, with lipid droplets contributing to proteostasis by sequestering misfolded proteins and serving as substrates for lipophagy. Below, we summarize how autophagy contributes to proteostasis in each of these paradigms.

### Autophagy and proteostasis in reduced IIS

*C. elegans* with reduced IIS, such as *daf-2* loss of function mutants*,* have extended lifespan and enhanced proteostasis. These effects are mediated by a network of stress-responsive transcription factors, including DAF-16/FOXO, HSF-1, SKN-1/NRF2, and HLH-30/TFEB^5^, ^100^, ^101^, ^102^, ^103^ (Figure 3). DAF-16/FOXO regulates stress resistance and metabolism, indirectly promoting autophagy by repressing *daf-15/RPTOR*, a component of mTORC1, a major autophagy inhibitor.^104^ HSF-1 enhances chaperone capacity and is required for proteostasis and thermotolerance in *daf-2* mutants.^105^ SKN-1/NRF2 activates detoxification and autophagy genes, including *atg-18* and *lgg-1/ATG8/GABARAP*.^106^ HLH-30/TFEB directly upregulates autophagy and lysosomal genes and is essential for *daf-2* longevity.^107^ Together, these transcription factors remodel autophagy, lysosomal function, and protein quality control to promote longevity.

Tissue-specific studies reveal that IIS-mediated longevity does not require autophagy uniformly. For example, *atg-18* is required in muscle but not intestine for full lifespan extension in *daf-2* mutants.^55^ This suggests that IIS-mediated longevity arises from autophagy activation in select tissues, particularly muscle, rather than organism-wide upregulation. At the same time, systemic signals modulate proteostasis across tissues. Neurons regulate intestinal stress responses and vice versa,^108^ while germline-to-soma signaling engages autophagy and lysosomal pathways to preserve muscle and intestinal proteostasis.^109^ Thus, IIS longevity reflects both tissue-autonomous autophagy requirements and organism-wide coordination of proteostasis.

Consistently, *daf-2* mutants show broad proteostasis improvement. Lifespan extension is accompanied by reduced paralysis and lower levels of disease-associated protein aggregation, including PolyQ, Aβ, and α-synuclein.^79^, ^105^, ^110^, ^111^, ^112^ Rather than uniformly enhancing aggregate clearance, IIS mutants modulate how aggregates are handled, often favoring sequestration into less toxic inclusion bodies (see “Autophagy-mediated inclusion body formation and aggregate compartmentalization”). Surprisingly, pulse-chase labeling revealed that *daf-2* mutants maintain proteome stability not through increased protein turnover, but via reduced rates of both protein synthesis and degradation, supported in part by trehalose accumulation.^113^ Trehalose has been reported to induce autophagy in mammalian systems,^114^ but the requirement of autophagy for this stabilization in *C. elegans* has not been tested. Importantly, IIS suppression can restore proteostasis even late in life. Auxin-inducible degradation of DAF-2 at day 20 of adulthood showed that late-life IIS inhibition extendsed lifespan, reinstates resistance to bacterial, heat, osmotic, and oxidative stress, and reduces aggregation of endogenous proteins such as PAB-1 and RHO-1.^115^ Recent work further demonstrates that degradation of DAF-2 in single tissues, including neurons or intestine, is sufficient at advanced ages to extend lifespan, restore resilience, and improve proteostasis, despite persistent mid-life pathologies.^115^ These findings highlight that IIS interventions act both locally and systemically to reactivate proteostasis, even in old animals.

Finally, HPK-1, the *C. elegans* ortholog of homeodomain-interacting protein kinases HIPK1–4, has been identified as a central integrator linking autophagy and chaperone pathways. In *daf-2* mutants, HPK-1 contributes to lifespan extension and proteostasis through two distinct mechanisms: it enhances chaperone expression via HSF-1 and independently promotes autophagy through the transcription factors PHA-4/FOXA and MXL-2/MLX, acting independently of HLH-30/TFEB.^116^ Overexpression of *hpk-1* delays polyQ aggregation and improves motor function and survival, while loss of *hpk-1* accelerates proteostasis collapse and reduces stress resistance, particularly in muscle tissue. Notably, *hpk-1* expression declines after reproduction ceases, suggesting that aging actively downregulates this protective mechanism. Together, these findings position HPK-1 as a key transcriptional node that integrates autophagy and chaperone pathways to support IIS-mediated longevity.

Collectively, evidence demonstrates that IIS longevity relies on selective and coordinated activation of autophagy, supported by transcriptional remodeling that sustains proteostasis across tissues. The ability of IIS suppression to restore proteostasis even late in life underscores its plasticity and firmly positions autophagy as a central mechanism linking stress resilience to lifespan extension.

### Autophagy and proteostasis in reduced mTORC1 signaling

mTORC1 inhibition is another conserved longevity pathway that promotes autophagy and proteostasis in *C. elegans* (Figure 3). Genetic inhibition of *let-363/*MTOR, *mlst-8* (a mTORC1 subunit), *daf-15/RPTOR*, as well as pharmacological inhibition using rapamycin robustly extends lifespan in *C. elegans*.^74^, ^117^ mTORC1 inhibition leads to the dephosphorylation of the autophagy initiation kinase UNC-51/ATG1/ULK1, activating it and enabling phagophore formation. mTORC1 inhibition enhances the nuclear translocation of HLH-30/TFEB, which activates autophagy and lysosomal genes.^107^ Autophagy is essential in this longevity paradigm, since knockdown of *bec-1/ATG6/BECN1*, *unc-51/ATG1/ULK1*, *hlh-30/TFEB*, and *atg-18* abolishes lifespan extension following mTORC1 inhibition.^107^, ^118^ Interestingly, RNAi knockdown of *mlst-8* produces tissue-specific effects with a reduction of polyQ aggregation in intestine and neurons, but paradoxically an increase in muscle.^74^ These findings provide further evidence that autophagy’s effects on proteostasis and longevity are highly context dependent. This is further illustrated by a study on SGK-1, a kinase downstream of the IIS pathway and mTORC2 that regulates mitochondrial homeostasis and stress resistance. In *sgk-1* mutants, elevated autophagy unexpectedly shortens lifespan due to increased mitochondrial permeability via the mitochondrial permeability transition pore (mPTP).^119^ Suppressing autophagy or blocking mPTP opening restores lifespan, suggesting that mitochondrial integrity dictates whether autophagy is protective or detrimental. Notably, mPTP opening also blocks autophagy-dependent lifespan extension in other paradigms, including DR and *glp-1* animals.

### Autophagy and proteostasis in DR

DR models, including *eat-2* mutants with reduced food intake, exhibit increased GFP:LGG-1/Atg8 puncta in hypodermal seam cells.^120^ Since autophagy genes and *hlh-30/TFEB* are required for the DR-induced lifespan extension, these data suggest that autophagy is enhanced in DR.^63^, ^73^, ^120^ In the intestine of *eat-2* mutants, GFP:LGG-1/Atg8 puncta are decreased, but further analysis shows enlarged lysosomal compartments and increased autolysosome numbers by day 7 of adulthood.^59^ RNAi against *vha-15*, which blocks lysosomal acidification, increases GFP:LGG-1/Atg8 puncta in *eat-2* animals,^63^ suggesting that the reduced puncta reflect increased autophagosome turnover, not reduced autophagy. Intestinal-specific autophagy genes *atg-18*, *lgg-1/ATG8/GABARAP*, and *lgg-2/ATG8/LC3* are required for the DR-induced lifespan extension. DR also reduces proteotoxicity in muscle, delaying paralysis in animals expressing polyQ or Aβ without reducing aggregate load.^121^ The transcription factor HSF-1 is required for this protective effect,^121^ implicating transcriptional responses such as chaperone induction or autophagy activation in proteostasis maintenance. However, the tissue in which *hsf-1* is required has not been defined. Furthermore, while DR improves organismal proteostasis, intestinal expression of aggregation-prone polyQ models has not been examined, and it is unknown whether autophagy is directly upregulated in muscle or whether improved clearance is mediated through inter-tissue signaling. Thus, whether DR acts through tissue-intrinsic autophagy or through systemic proteostasis regulation remains an open question.

### Autophagy and proteostasis in *glp-1* animals

The germline acts as a systemic regulator of lifespan and proteostasis, with autophagy playing a central role.^122^ In *C. elegans*, removal of the germline, via mutation in the Notch family receptor GLP-1, or laser ablation, extends lifespan and improves proteostasis across somatic tissues^45^, ^122^, ^123^, ^124^ (Figure 3). Lifespan extension in *glp-1* mutants requires autophagy genes and the transcription factors DAF-16/FOXO, PHA-4/FOXA HLH-30/TFEB, and HSF-1.^45^, ^107^, ^124^, ^125^ Germline-mediated longevity also involves lipid metabolism via DAF-16/FOXO-dependent upregulation of the lysosomal lipase LIPL-4, which is required for autophagy induction and lifespan extension in *glp-1* animals.^125^ Germline removal enhances not only lipid metabolism and autophagy but also UPS activity. In *glp-1* mutants, proteasome function is increased through DAF-16/FOXO-dependent upregulation of *rpn-6.1/PSMD11*, a regulatory subunit.^45^ Reducing *rpn-6.1/PSMD11* suppresses lifespan extension and worsens neuronal polyQ67 and polyQ40-induced motility defects, while its overexpression improves motor function and decreases insoluble polyQ67 aggregates.^45^ *glp-1* mutants also show greater resistance to proteotoxic stress, including heat, with elevated expression of *hsp-70*, *hsp-16.11/HSPB1*, *hsp-16.2/HSPB1,* dependent on DAF-16/FOXO.^124^ Germline removal reduces paralysis in mutants expressing ts misfolding protein *unc-54(ts)*, stabilizes cytoskeletal structures, and lowers polyQ aggregation in muscle.^124^ These protective effects require transcription factors PHA-4/FOXA, DAF-16/FOXO, HLH-30/TFEB, and HSF-1.^107^, ^124^, ^125^ In sum, these factors coordinate stress response and degradative pathways to promote somatic proteostasis and lifespan. Although autophagy is required for lifespan extension in *glp-1* mutants, its direct role in reducing protein aggregation remains unclear and needs further study. Recent work also shows that germline proteostasis influences somatic stress responses via HSF-1 and Wnt signaling, suggesting that the germline helps coordinate organism-wide proteostasis.^94^ While this study emphasized mitochondrial roles, autophagy may also contribute to somatic proteostasis downstream of germline signaling. In parallel, piwi-interacting-RNA-regulated Hedgehog ligands from the germline have been shown to shorten lifespan in part by suppressing the mitochondrial unfolded protein response (UPR^mt^) in somatic tissues.^126^, ^127^ These pro-aging signals imply that the germline may constrain somatic autophagy, prioritizing reproduction over long-term cellular maintenance. Whether autophagy is directly involved in these pathways remains to be determined.

### Autophagy and proteostasis in mild mitochondrial dysfunction

Mild mitochondrial dysfunction is another longevity paradigm in *C. elegans*, in which partial inhibition of the electron transport chain (ETC) triggers adaptive stress responses that extend lifespan (Figure 3). Mutations or RNAi targeting ETC components, such as *isp-1/UQCRFS1*, *atp-3/ATP5O*, *nuo-2/NDUFS3*, *cco-1/COX5B*, *frh-1/NDUFAB1*, and *clk-1/COQ7*, extend lifespan by inducing low-level mitochondrial stress*.*^128^, ^129^, ^130^ However, lifespan extension depends on dose, developmental timing, and tissue specificity of the perturbation. A key protective mechanism in this paradigm is activation of the UPR^mt^, driven by the transcription factor ATFS-1. ATFS-1 upregulates mitochondrial chaperones (*hsp-6/HSPA9*, *hsp-60/HSPD1*), proteases, mitochondrial import, and antioxidant genes to restore mitochondrial quality control.^131^ Mild mitochondrial stress also induces mitophagy, the selective clearance of damaged mitochondria. In *isp-1* mutants, mitophagy is required for full lifespan extension, as RNAi knockdown of *pink-1*, a key mitophagy regulator, partially suppresses their longevity.^132^ This pathway also improves proteostasis. For example, in *nuo-6* mutants, neuronal ETC disruption activates UPR^mt^ and reduces protein aggregation in the intestine, demonstrating cell-nonautonomous regulation of proteostasis.^133^ In addition, glial activation of UPR^mt^ via overexpression of the histone demethylate, *jmjd-1.2*, reduces protein aggregation in neurons in a cell-nonautonomous manner.^134^ However, direct evidence that mild mitochondrial dysfunction reduces polyQ or Aβ aggregation is limited. The specific role of autophagy beyond mitophagy in this paradigm remains underexplored; however, the requirement for *hlh-30/TFEB* in *clk-1* RNAi-mediated lifespan extension suggests broader autophagic remodeling may contribute to longevity in mitochondrial mutants.^107^

## Stress-induced autophagy in proteostasis maintenance in *C. elegans*

While basal autophagy is important for routine cellular maintenance, it is rapidly upregulated in response to proteotoxic stress such as nutrient deprivation, oxidative stress, or accumulation of misfolded proteins.^73^, ^135^, ^136^, ^137^ This stress-induced autophagy helps preserve proteostasis by clearing damaged organelles and aggregated proteins and often acts in a tissue-specific manner. One major protective pathway is the heat shock response, mediated by HSF-1, which induces the expression of *hsp-1/HSP70* and *daf-21/HSP90*, which recognize and stabilize misfolded proteins.^138^, ^139^ HSF-1 overexpression enhances clearance of polyQ aggregates.^105^ Autophagy genes are upregulated in HSF-1-overexpressing animals and knockdown of autophagy genes (*unc-51/ATG1/ULK1, bec-1/ATG6/BECN1, lgg-1/ATG8/GABARAP*) abrogates improvements in neuronal and intestinal polyQ models.^65^ Hormetic heat shock also strongly induces nuclear translocation and activation of HLH-30/TFEB, leading to transcription of autophagy-related genes acting in each step of the autophagy process.^65^, ^107^ This activation extends lifespan and improves proteostasis, both of which require autophagy genes *bec-1/ATG6/BECN1* and *sqst-1/p62*.^28^, ^65^

Oxidative stress similarly activates autophagy via SKN-1/NRF2, which regulates antioxidant and autophagy gene expression.^102^ SKN-1/NRF2 protects against ROS-generated damage by promoting clearance of oxidized proteins and dysfunctional mitochondria, extending lifespan.^102^, ^140^ These effects require core autophagy genes such as *atg-18* or *lgg-1/ATG8/GABARAP*. However, not all SKN-1/NRF2 activity enhances autophagy, since a gain-of-function *skn-1/NRF2* mutant shifts transcription toward immune response genes, highlighting isoform- and context-specific effects.^141^ During prolonged oxidative stress, SKN-1/NRF2 can downregulate autophagy by repressing *aak-2*/AMPK expression, providing a negative feedback mechanism that prevents excessive autophagic activity.^142^

The unfolded protein response in the endoplasmic reticulum (UPR^ER^) is triggered by ER stress and initiated by three conserved stress sensors: IRE-1, ATF-6, and PEK-1/PERK. IRE-1 activates the transcription factor XBP-1 via mRNA splicing; PEK-1/PERK inhibits translation and induces stress responses; and ATF-6 functions both as a sensor, and upon cleavage, as a transcription factor that upregulates ER chaperones.^143^ Autophagy can be induced downstream of UPR^ER^ to clear misfolded proteins and restore ER homeostasis. In *C. elegans*, loss of the chromatin regulator HPL-2/HP1 enhances the IRE-1–XBP-1 branch and increases GFP:LGG-1/Atg8 puncta in hypodermal seam cells. This correlates with improved survival under ER stress, which is abolished by *lgg-1/ATG8/GABARAP* RNAi, demonstrating that autophagy is required for this protection.^144^ Lipid stress, induced by impaired phosphatidylcholine synthesis, leads to the induction of the IRE-1–XBP-1 branch of the UPR^ER^, which in turn induces autophagy genes such as *bec-1/ATG6/BECN1* and *lgg-1/ATG8/GABARAP,* as well as lipolysis genes. GFP:LGG-1/Atg8 puncta are increased and lipid droplets decreased in the intestine in an *ire-1*-dependent manner, confirming the requirement of the IRE-1–XBP-1 axis for autophagy induction.^145^

Activation of the UPR^mt^ induces mitochondrial chaperones like *hsp-6*/HSPA9 and *clpp-1*^146^ to restore mitochondrial homeostasis, but accumulating evidence suggests that autophagy also contributes to proteostasis during mitochondrial stress. One example comes from perillaldehyde, a natural compound that induces mild mitochondrial stress in *C. elegans*. Treatment with perillaldehyde reduces polyQ aggregation in neurons and muscle, extends lifespan, and improves healthspan, effects linked to activation of AAK-2/AMPK signaling and UPR^mt^-mediated serotonin-dependent, cell-non-autonomous signaling.^147^ These findings highlight how mitochondrial stress can enhance proteostasis through both local and systemic mechanisms. Mitochondrial stress can also be triggered by cytosolic metabolic disruption; for example, knockdown of the pentose phosphate pathway enzyme *tald-1/TALDO1* impairs mitochondrial function and increases oxidative stress, which activates HLH-30/TFEB to enhance autophagy and improve proteostasis.^148^ This indicates that cytosolic redox imbalance can trigger mitochondrial quality control pathways, including autophagy, even in the absence of direct ETC inhibition. Finally, inhibition of mitochondrial translation alone is sufficient to extend lifespan and induce autophagy through HLH-30/TFEB, even in the absence of UPR^mt^, suggesting that lysosomal biogenesis and autophagy are sufficient to confer mitochondrial protection.^149^ Notably, this effect was enhanced by disrupting mitochondrial dynamics, revealing a compensatory crosstalk between mitochondrial morphology and autophagy pathways.

Overall, these findings underscore autophagy as a central effector of stress adaptation, acting across multiple cellular pathways to preserve proteostasis and ensure organismal resilience under conditions of environmental and intracellular stress.

## Autophagy-mediated inclusion body formation and aggregate compartmentalization

Protein aggregation arises when misfolded or partially folded proteins self-associate into oligomers and larger complexes. These aggregates may be amyloid-like or amorphous but are generally insoluble and resistant to degradation.^150^ They vary in size and composition, ranging from small, soluble oligomers (<500 nm), which are considered more cytotoxic, to larger, more inert inclusion bodies (>1.5 µm).^151^ In *C. elegans* expressing human Aβ, *daf-2* RNAi reduces paralysis without decreasing large aggregate levels, while loss of DAF-16/FOXO reduces large aggregate levels but paradoxically increases paralysis, and loss of HSF-1 increases both large aggregate accumulation and paralysis.^111^ This suggests that smaller, soluble oligomers are more toxic than larger inclusions and that cellular mechanisms may shift aggregation toward less harmful states. However, the precise role of autophagy in modulating these opposing protective pathways remains unclear.

Inclusion bodies are protective structures that sequester aggregates and can form via direct recruitment of misfolded proteins to nucleation sites or by the coalescence of smaller aggregates. Inclusion bodies are thought to function as protective compartments that isolate potentially harmful proteins from the surrounding cytoplasm.^67^, ^71^ Selective autophagy receptors like p62/SQSTM1 scaffold ubiquitinated proteins into inclusions by oligomerizing through its PB1 domain. In HeLa cells p62/SQSTM1 localizes to inclusion bodies that co-stain with both ubiquitin and LC3/Atg8. Importantly, both the PB1 domain (for oligomerization) and the UBA domain (for ubiquitin binding) are required for the formation of these cytoplasmic structures.^29^, ^152^ Recent studies have shown that this scaffolding activity is driven by liquid-liquid phase separation, whereby p62/SQSTM1 forms dynamic condensates with ubiquitinated proteins to cluster misfolded substrates and promote their delivery to autophagosomes.^29^, ^32^ In *C. elegans*, overexpression of *sqst-1*/p62 leads to the induction of GFP:LGG-1/Atg8 puncta that are exceptionally large,^28^ raising the possibility that these represent inclusion-like structures. Notably, in *C. elegans*, overexpression of SQST-1/p62 at 25 °C leads to its progressive accumulation in large inclusions, coinciding with reduced lifespan,^33^ as discussed in “Selective Autophagy: Aggrephagy.” This suggests that SQST-1-driven condensates or inclusion-like structures can themselves exert detrimental effects when not properly balanced with degradation capacity.

In *C. elegans* disease models of ALS caused by mutations in the RNA-binding protein FUS, cytoplasmic FUS mislocalization impairs basal autophagy in neurons.^93^ This leads to SQST-1/p62 accumulation in neurons and neuromuscular dysfunction. Loss of *sqst-1/p62* suppresses these functional deficits without restoring neuronal autophagy, suggesting that SQST-1 may contribute to pathogenesis through aggregate formation.^93^ Notably, in mammalian ALS-FUS neurons, p62 co-aggregates with mutant FUS, pointing to a direct role for p62 in the formation or stabilization of protein aggregates. These findings support a growing view that autophagy components, including selective receptors like p62, can influence proteostasis not only by mediating degradation but also by modulating aggregate dynamics, spatial compartmentalization, and stress signaling.

In mammals, a specialized subtype of inclusion body is the aggresome, which forms at the microtubule-organizing center via dynein-mediated retrograde transport (Figure 1). Aggresomes concentrate aggregates for autophagic clearance and are often surrounded by a vimentin cage,^153^, ^154^, ^155^ a dense cytoskeletal shell composed of intermediate filaments that spatially restrict aggregates and insulate surrounding cellular components. Aggresomes can serve as autophagy substrates and are scaffolded by autophagy-linked FYVE protein ALFY, which tethers p62-bound cargo to autophagic membranes.^156^ While ALFY has no known *C. elegans* ortholog, aggresome-like structures have been observed using fluorescently tagged intermediate filaments IFD-1 and IFD-2.^157^ They are large, perinuclear protein aggregates that form in mechanosensory neurons when misfolded or ubiquitinated proteins are transported along microtubules to the microtubule-organizing center for sequestration and eventual degradation, likely by autophagy. In animals with impaired autophagy, either through *epg-9/RUBCN* mutation or treatment with the autophagy inhibitor Spautin-1, aggresome number and size significantly increase. Moreover, colocalization of the aggresome marker RFP:IFD-2 with the selective autophagy receptor SQST-1/p62:GFP indicates that autophagy components are present at neuronal aggresomes,^157^ but whether this reflects targeting for autophagic degradation, promotion of aggregate formation, or spatial organization through mechanisms such as phase separation needs further study.

In parallel, chaperone-driven inclusion formation offers an alternative spatial quality control mechanism. Specific small heat shock proteins (sHSPs) in *C. elegans*, enriched for aromatic and methionine residues, act as sequestrases that drive protective inclusion formation under proteotoxic stress. These sHSPs rescue yeast mutants lacking sequestration capacity, are upregulated in *daf-2* mutants, and contribute to lifespan extension.^158^ Consistently, proteome-wide showed that long-lived *daf-2* mutants actually accumulate more insoluble proteins than wild type, but sequester them into numerous small, sHSP-enriched aggregates, whereas *daf-16/FOXO* mutants show premature proteostasis collapse.^36^ The activity of these sHSPs complements autophagy, emphasizing that multiple conserved systems coordinate aggregate compartmentalization to maintain proteostasis.

These insights illustrate how cells leverage inclusion body formation, selective autophagy receptors, and aggresome dynamics as coordinated spatial strategies to contain, manage, and clear toxic protein aggregates, preserving proteostasis and organismal health.

## Noncanonical roles of autophagy in proteostasis

Beyond lysosomal degradation, autophagy-related proteins also participate in non-canonical processes, including secretion of intracellular material and inter-tissue signaling.^7^, ^10^ These emerging roles highlight the versatility of autophagy components in coordinating organismal proteostasis.

### Secretory autophagy

In *C. elegans*, misfolded and aggregation-prone proteins can be transmitted between tissues, revealing that cellular mechanisms exist for the intercellular transfer of toxic protein species. For example, expanded polyQ proteins expressed in *C. elegans* pharyngeal muscle can be transmitted to adjacent neurons, as shown by bimolecular fluorescence complementation. This age-associated transfer induces neurodegeneration, pharyngeal dysfunction, and reduced lifespan,^159^ suggesting that proteotoxic stress can propagate across tissues via direct contact. Additional evidence for intercellular transmission of aggregation-prone proteins comes from a *C. elegans* model expressing the yeast prion domain NM of Sup35 in body-wall muscle. In this model, NM aggregates due to expanded oligopeptide repeats in the Q/N-rich N-terminus, which cause developmental delay, embryonic and larval arrest, and widespread tissue degeneration.^160^ Interestingly, these toxic aggregates co-localize with autophagy-related vesicles, which appear to mediate cell-nonautonomous spread to adjacent tissues, including neurons and epidermis. This is accompanied by mitochondrial fragmentation and dysfunction, particularly in non-expressing cells, and a systemic collapse of proteostasis. These findings suggest that autophagy-related trafficking pathways may inadvertently facilitate prion-like propagation of misfolded proteins across tissues *in vivo*.

In both human cells and *C. elegans,* autophagy proteins can facilitate unconventional secretion of cytosolic proteins, away from lysosomal degradation toward the plasma membrane or multivesicular bodies for extracellular release (Figure 1). This pathway facilitates secretion of cytokines such as IL-1β and proteotoxic aggregates, including amyloid-β and α-synuclein.^161^, ^162^, ^163^ In neurons harboring PD-associated LRRK2 mutations, which impair canonical autophagic degradation, secretory autophagy and exosome release are upregulated as compensatory mechanisms to expel undegraded autophagic cargo and maintain proteostasis.^164^

In *C. elegans*, a striking example of noncanonical autophagic disposal is the formation of “exophers,” large vesicles that bud off from neurons under proteotoxic stress and carry misfolded proteins like polyQ aggregates^165^ (Figure 2a). Exopher production increases upon the neuronal-specific reduction of early autophagy genes, except *atg-16.2*,^7^ whose WD40 domain is specifically required for exopher formation in mechanosensory ALM neurons.^7^ These vesicles are engulfed via phagocytosis by neighboring hypodermal cells for degradation,^166^ a process that produces a characteristic “starry night” pattern. Although the precise mechanism is still unclear, the requirement of late-stage autophagy gene *cup-5*, which is required for proper degradation of the autolysosome,^167^, ^168^ and co-localization of LGG-1/Atg8/GABARAP, suggests that phagocytosed exophers may fuse with autophagosomes in hypodermal cells to complete degradation.^166^, ^169^ This supports a model where reduced autophagy in neurons enhances exopher-mediated disposal, highlighting a compensatory, neuroprotective role for secretory autophagy in maintaining proteostasis.

### Inter-tissue regulation of proteostasis

While proteostasis is traditionally considered strictly cell-autonomous, *C. elegans* studies show it is coordinated across tissues through inter-organ signaling. A key example is UPR^ER^-mediated communication: expression of the spliced and active form of transcription factor XBP-1 (XBP-1s) in neurons or glia activates stress responses and enhances proteostasis in peripheral tissues. Neuronal XBP-1s reduces muscle and intestine polyQ aggregation, while glial XBP-1s activates HLH-30/TFEB in the intestine, inducing autophagy, lipid catabolism, stress resistance, and longevity.^170^, ^171^ Neuronal UPR^ER^ also triggers lipophagy in the intestine, promoting ER remodeling and lipid depletion.^172^ Finally, some autophagy components, including *bec-1/ATG6/BECN1*, are partially required for the lifespan extension found in these animals.^171^ In parallel, reduction of mitochondrial ETC activity in either neurons or the intestine via RNAi knockdown of *cco-1/COX5B* extends lifespan by inducing the UPR^mt^. Interestingly, neuronal depletion of *cco-1/COX5B* induces a cell-nonautonomous increase of UPR^mt^ reporter *hsp-6p:GFP* in the intestine, suggesting that neurons can release “mitokine” signals that transmit mitochondrial stress to distal tissues to coordinate systemic proteostasis and longevity.^133^ In addition, UPR^mt^ activation solely in glia was sufficient to drive reduction of protein aggregates in neuronal cells.^134^ These findings demonstrate that UPR^ER^ and UPR^mt^ signaling from the nervous system can drive inter-tissue regulation of proteostasis by triggering multiple peripheral programs, including chaperone expression and metabolic remodeling, yet it remains unclear whether autophagy also contributes to UPR^mt^-mediated benefits, as has been shown for UPR^ER^.

HLH-30/TFEB plays a central role in systemic stress responses and longevity regulation through cell-nonautonomous mechanisms. Neuronal HLH-30 overexpression promotes heat stress resistance by modulating neurotransmission via the protein W06A11.1, which drives mitochondrial fragmentation in peripheral tissues,^173^ highlighting that neurons can coordinate protective responses in distant cells without directly inducing autophagy gene transcription. Inter-tissue regulation also extends to selective autophagy. Neuronal overexpression of the autophagy receptor SQST-1/p62 extends lifespan in *C. elegans*.^28^ This suggests that boosting neuronal selective autophagy can have organism-wide benefits. Whole-body overexpression of SQST-1/p62 improves the mobility of neuronal polyQ aggregates, suggesting enhanced degradation or altered aggregate properties, but whether neuronal-specific SQST-1/p62 overexpression can influence proteostasis in peripheral tissues through cell-nonautonomous effects remains an intriguing question.

The intestine also regulates systemic proteostasis through secreted microRNAs. The age-associated increase in intestinal *mir-83/miR-29*, induced by HSF-1, leads to its transport to other tissues, including muscle, potentially via extracellular vesicles, where it suppresses *cup-5*.^174^ This suppresses autophagy and increases polyQ aggregation in the intestine and muscle. Notably, mutation of *mir-83* restores autophagy in multiple tissues, highlighting how intestinal microRNA signaling modulates organismal proteostasis.

Cumulatively, these findings reveal that autophagy and proteostasis in *C. elegans* are regulated by coordinated inter-tissue communication, with signals from neurons, glia, and intestine shaping organism-wide responses.

## Conclusions

Autophagy, as a key member of the proteostatic network, plays a central and multifaceted role in proteostasis at both the cellular and systemic levels to maintain protein quality and in promoting stress resistance, particularly during aging and in response to stress stimuli. Research in *C. elegans* has provided valuable insight into the molecular mechanisms which govern the autophagy process and has highlighted their role clearing misfolded proteins, degrading aggregates, and supporting longevity paradigms associated with improved proteostasis. *C. elegans* has allowed for tissue-specific investigation of autophagy and proteostasis in various tissues, including neurons, intestine, muscle, and germline. Although proteostasis declines with age in both somatic and germline tissues, autophagy may become selectively preserved, compartmentalized, or dysregulated across different tissue types. This highlights the need to define tissue-specific drivers and outcomes of autophagy function and dysfunction, particularly in the context of aging and stress. Emerging evidence also suggests that proteostasis failure can propagate across tissues, highlighting the need to understand how autophagy is regulated at the organismal level to maintain systemic homeostasis.

Despite its protective roles, autophagy is not uniformly beneficial across tissues or contexts. In *C. elegans*, certain pro-autophagic interventions reduce aggregate burden in the intestine but exacerbate aggregation in body-wall muscle. Similarly, in mammals, both excessive and insufficient autophagy in skeletal muscle accelerate muscle-wasting phenotypes. These findings underscore that autophagy in aging muscle, where proteostasis failure contributes to sarcopenia, is a finely tuned process that can become detrimental when dysregulated. Such context-dependent effects highlight the need for tissue-specific strategies when targeting autophagy for therapeutic purposes.

Further, emerging evidence suggests that autophagy extends beyond canonical intracellular degradation to encompass noncanonical functions, including aggregate compartmentalization, secretory pathways, and inter-tissue communication. For example, stress signals from neurons or the intestine can trigger autophagy in distal tissues, and secreted miRNAs or exophers may modulate proteostasis organism-wide. These noncanonical roles, together with observations of autophagy’s involvement in spatial quality control and inter-organ signaling, position autophagy as a hub for systemic coordination of proteostasis. Future studies should also investigate how cytoskeletal dynamics, especially age-related actin remodeling, influence autophagic flux, spatial proteostasis, and aggregate handling, given its ties to longevity pathways. Moreover, the complex crosstalk between autophagy and the UPS further adds to this regulatory landscape, challenging the assumption that these systems function purely redundantly and highlighting the need to study them in tissue- and context-specific detail.

Importantly, conserved longevity paradigms highlight how sustained or enhanced autophagy supports proteostasis and healthy aging. Understanding how autophagy contributes to proteostasis in specific longevity contexts, such as reduced IIS, DR, *glp-1*, and hormetic stress response (eg, mitochondrial stress and heat stress), will be essential for translating these findings across systems. In addition, metabolic pathways, including lipid droplet dynamics, have emerged as active participants in proteostasis: they can serve as protective reservoirs for misfolded proteins and engage lipophagy to couple lipid metabolism with protein quality control. Selective autophagy receptors have been identified for protein aggregates (SQST-1/p62) and damaged mitochondria (DCT-1/BNIP3), while receptors that directly target lipid droplets remain unknown. Together with stress-responsive transcription factors, these pathways coordinate protective effects under stress and in disease models such as HD, AD, PD, and ALS. These aggregation models thus demonstrate that autophagy is a critical, tissue-specific regulator of proteostasis in *C. elegans*, capable of mitigating toxic protein accumulation in both physiological and disease contexts. Understanding how to selectively enhance autophagy’s beneficial roles without disrupting homeostasis represents a major challenge and opportunity for therapeutic development.

## Funding and support

CML is funded by the California Institute for Regenerative Medicine Predoctoral Fellowship. CK is supported by NIA grant R01AG083373. RHS is supported by NIA grant R01AG079806.

## CRediT authorship contribution statement

**Caitlin M. Lange:** Writing – review & editing, Writing – original draft. **Ryo Higuchi-Sanabria:** Writing – review & editing. **Caroline Kumsta:** Writing – review & editing, Writing – original draft. CML conceived and wrote the review, which was co-written by RHS and CK. CML, RHS, and CK contributed to collect and sort references. CML and CK designed the figures. All authors contributed to editorial changes in the manuscript and reference curation. All authors read and approved of the final manuscript. All authors have participated sufficiently in the work and agreed to be accountable for all aspects of the work.

## Declaration of Generative AI and AI-Assisted Technologies in the Writing Process

ChatGPT-4o was used as a thesaurus and for minor grammatical refinement during the drafting of this article.

## Declaration of Interest

The authors declare the following financial interests/personal relationships, which may be considered as potential competing interests: Caroline Kumsta reports financial support was provided by the National Institute on Aging Division of Aging Biology. Ryo Higuchi-Sanabria reports financial support was provided by the National Institute on Aging Division of Aging Biology. Caitlin M Lange reports financial support was provided by the California Institute for Regenerative Medicine. If there are other authors, they declare that they have no known competing financial interests or personal relationships that could have appeared to influence the work reported in this paper.

## Data Availability

No data was used for the research described in the article.
